# A Day in the Life of a Ugandan Healthcare Provider: Managing Neonatal Tetanus

**DOI:** 10.4269/ajtmh.24-0774

**Published:** 2025-04-08

**Authors:** Kaitlyn M. Pereira, Edrin Jjuuko

**Affiliations:** ^1^ Department of Emergency Medicine, Medical University of South Carolina, Charleston, South Carolina;; ^2^ OneWorld Health, Kampala, Uganda

My day, along with that of Dr. Brian, the physician on duty, began with a referral from Kinyara Health Center III after morning devotion. The intake sheet indicated that a mother had brought her newborn son in, distressed by his high temperature, convulsions, and poor feeding. Upon arrival, the infant was burning with a fever of 40°C, although his blood glucose levels were normal. His initial diagnosis was neonatal sepsis, a common yet dangerous condition in our region. We started him on ampicillin and cefotaxime, but something was amiss. The infant’s chest was withdrawing with each breath, a sign of respiratory distress, and his convulsions were intense enough to warrant rectal diazepam, necessitating transfer to a higher level of care. His arrival occurred during my first week in Uganda, marking the beginning of my 1-month global health trip. Back in the United States, I had never witnessed the extent of his tonic–clonic convulsions. Puzzled by his presentation, I looked to Dr. Brian for answers, and together we determined that we needed more information from his mother about the birthing process.

Upon arrival at our hospital, Masindi Kitara Medical, the infant’s history unfolded. He was born at home through a spontaneous vaginal delivery after a prolonged rupture of membranes. The birth was not attended by a skilled professional, and his umbilical cord had been cut with an unsterilized knife and tied with a thread. Despite crying immediately after birth, the infant fell ill 7 days later, just as the umbilical cord fell off. The site appeared swollen and infected, a classic scenario for umbilical sepsis, or omphalitis. The infant’s vitals were very worrisome: a fever of 39.2°C, a heart rate of 172 bpm, a respiratory rate of 40, and oxygen saturation at 92%. His abdomen was swollen, his muscle tone increased, and he displayed weak strength—signs of a severe infection. We began treatment with oxygen support, intravenous phenobarbital for seizures, and continued antibiotics; however, the constellation of symptoms did not completely fit the picture of omphalitis because seizures do not usually occur with this diagnosis. Feeling helpless because of the lack of resources, Dr. Brian reassured me that we were providing the best care possible. I was rattled by many thoughts: germinal matrix hemorrhage or an intracranial hemorrhage due to the prolonged birth, febrile seizures (although I felt that something more sinister was occurring based on his age and persistent increased tone), and finally meningitis, for which he was luckily already receiving treatment. I wondered if a lumbar puncture was indicated, but because I was new and unsure of its feasibility, I did not express my concerns to Dr. Brian.

Two days later, his persistent tonic–clonic seizures emerged, triggered by simple touches, lasting less than 1 minute each. This new development prompted a thought in Dr. Brian’s mind. Combing through all the pertinent information from this case—an unsterile knife, an infected umbilical cord, delayed presentation, and now tactile-triggered seizures—led us to reconsider our diagnosis: neonatal tetanus, a condition I have never witnessed because of widespread vaccinations. Dr. Brian referred to the Uganda Clinical Guidelines for appropriate treatment because the condition is uncommon in Uganda, with fewer than 100 cases reported annually according to the WHO/United Nations International Children’s Emergency Fund Joint Reporting Form on Immunization. We moved the infant to a dark room to reduce stimulation, tailored the antibiotic regimen for neonatal tetanus, including metronidazole, and started diazepam every 4 hours to control the spasms. Given the strong suspicion of tetanus, we administered tetanus immunoglobulin, a crucial step in his care. Upon this discovery, an overwhelming feeling of dismay spread across the entire treatment team because of the high morbidity and mortality.

The next few days were critical. Despite our best efforts, the infant continued to have seizures and spasms, and his face became puffy, indicating systemic involvement. His jaw locked, and tactile stimulation caused severe spasms, a classic sign of tetanus. The spasms became so intense that his body would arch backward in opisthotonus, a grim manifestation of the disease that I had only seen in medical books. We alternated between diazepam and phenobarbital every 3 hours, trying to find a balance that would ease his suffering. Oral rehydration was impossible because of his locked jaw and continued increased muscle tone. Saliva would seep out of his mouth and nose continuously, requiring suctioning every couple of minutes. Aspiration was a significant concern, but our clinic did not have the resources to place the patient on a ventilator. We considered transferring him to a hospital in Kampala, Uganda’s capital, but ultimately decided against it because of the cost for the family and our hope for improvement in the coming days.

The sixth day brought a setback—his fever returned, and spasms occurred even without stimulation. We increased his medications and continued our treatment protocol, including tetanus immunoglobulin, hoping to see improvement. At this stage, I checked on the infant multiple times a day because I lived on the hospital grounds. I wondered if the family understood the extent of his disease. They spoke a dialect from a nearby village that only a few of our nurses could understand. I felt disheartened by his case because there was nothing else I could do or provide. Even if I had all the resources available, the survival rate would still be minimal. Finally, 2 days later, the fever subsided, and the spasms began to decrease. The infant’s muscles relaxed enough for him to cry again after a week, which was a small victory, but a sign of progress. On the 16th day, he attempted to suckle, and we knew the tide was turning. Hope was on the horizon, and transfer was no longer needed.

The next day marked a turning point: his condition stabilized enough to hold antibiotics and antispasmodics. That night, he slept without spasms for the first time since he was admitted. After 21 days of intensive care, the infant could finally breastfeed again, a testament to his resilience and the effectiveness of our care. Approximately 4 months later, the infant had a follow-up appointment and was successfully meeting all his developmental milestones—a welcome discovery that brought smiles to my face and to Dr. Jjuuko, Dr. Brian’s medical supervisor, who had followed and continues to follow his condition at Masindi Kitara Medical. Upon reflection, we both gained significant knowledge about neonatal tetanus and learned that the human body can endure more than we can imagine.

As I reflect on the past few weeks, I am reminded of the fragility of life and the critical importance of access to skilled birth attendants and clean delivery practices. Neonatal tetanus is a preventable condition, yet we find ourselves fighting to save a life that could have been spared this suffering. Our work is far from over, but today, this little one’s victory is our shared success. Education is vital, and simple instructions on how to cut the umbilical cord properly could have made all the difference. This case underscores the importance of community outreach and involvement.

Moving forward, I know that the experiences of this day will shape my approach to care and advocacy. We must do more to prevent such cases from ever reaching our doors. This serves as a reminder that although we can save lives within the walls of our hospital, the real work of saving lives often begins with prevention in the communities we serve.

